# Genomic deletion of TLR2 induces aggravated white matter damage and deteriorated neurobehavioral functions in mouse models of Alzheimer’s disease

**DOI:** 10.18632/aging.102260

**Published:** 2019-09-11

**Authors:** Chao Zhou, Xiaoyu Sun, Yuting Hu, Jiaxing Song, Shuyu Dong, Delian Kong, Yuqiao Wang, Xiaodong Hua, Jingjing Han, Yan Zhou, Guoliang Jin, Xinxin Yang, Hongjuan Shi, Zuohui Zhang, Fang Hua

**Affiliations:** 1 Department of Neurology, The Affiliated Hospital of Xuzhou Medical University, Xuzhou, China; 2 Institute of Neurological Diseases, Xuzhou Medical University, Xuzhou, China; 3 Department of Neurology, Xuzhou Central Hospital, Xuzhou, China; 4 Augusta University/University of Georgia Medical Partnership, Athens, GA 30606, USA; 5 Medical College of Georgia, Augusta University, Augusta, GA 30606, USA

**Keywords:** TLR2, Alzheimer’s disease, MRI, white matter damage, neurobehavioral function

## Abstract

Toll-like receptor-2 (TLR2), a member of the TLR family, plays an important role in the initiation and regulation of immune/inflammation response, which is a critical mechanism underlying Alzheimer’s disease (AD). To clarify the role of TLR2 in the pathological process of AD, in the present study, TLR2 knockout plus APPswe/PSEN1dE9 transgenic mice (AD-TLR2KO) were generated. Neurobehavioral tests and brain MRI scan were conducted on mice at the age of 12 months. Additionally, neuron loss was evaluated using NeuN staining. Amyloid β protein (Aβ), glial fibrillary acidic protein (GFAP), endogenous ligands for TLR2, and the activation of downstream signaling of TLR2 in mouse brains were detected by immunohistochemistry and Western blots. The results demonstrated that TLR2 deficit induced learning disabilities, decreased spontaneous activity, increased anxiety and depression, and led to white matter damage (WMD), brain atrophy, loss of neurons, and glial activation. Moreover, TLR2 deficit aggravated impaired neurobehavioral functions and WMD in AD mice, but did not affect the Aβ deposition in mouse brains. Our data indicate that the genomic deletion of TLR2 impairs neurobehavioral functions, induces WMD and brain atrophy, and increases the activation of astrocytes, which in turn aggravate the symptoms of AD through a non-Aβ mechanism.

## INTRODUCTION

Alzheimer’s disease (AD) is a neurodegenerative disease typified by chronic inflammation and neuronal loss in the brain [1, 2]. In patients with familial Alzheimer’s disease (FAD), mutations in the APP gene, PSEN1 gene, and PSEN2 gene were found. In addition, other candidate genes associated with AD were also identified, of which the polymorphic apolipoprotein E (apoE) gene was reported to be the most related [3]. Due to these genetic mutations, the deposition of Aβ and the hyperphosphorylation of the tau-protein appear in the brain [4, 5], inducing the loss of neurons, the activation of astrocytes, and the hyperactivation of microglia cells [6, 7]. Activated microglia and astrocytes release pro-inflammatory cytokines, leading to inflammatory responses, which are involved in not only neuronal death and neurofibrillary tangle formation but also in Aβ clearance and neuroregeneration [8]. Substantial evidence has demonstrated that inflammation plays a key role in the pathological processes of AD [9]. Excessive inflammation associated with the deposition of Aβ and the hyperphosphorylation of the tau-protein results in neuronal loss and white matter damage (WMD) [10–12]. On the other hand, moderate inflammation is helpful for eliminating the deposition of Aβ and for neuroregeneration [13]. The mechanisms underlying the regulation and modulation of inflammation in AD brains are, however, unclear at present.

Toll-like receptors (TLRs) are a family of type-1 transmembrane receptors. TLRs, possessing the toll/ interleukin-1 receptor (TIR) domain and leucine-rich repeat (LRR) motifs, regulate host defensive response via the myeloid differentiation primary response 88 (MyD88)-dependent pathway and/or the MyD88-independent signaling pathway [14]. Activated by ligands, TLRs recruit serial downstream kinases, leading to the activation of nuclear factor kappa B (NF-kB) and/or interferon regulatory factor 3 (IRF3) and resulting in the release of pro-inflammatory factors and anti-inflammatory factors [15]. TLRs were found to be expressed in neural precursor cells, neurons, and glial cells, and are involved in the immune functional maturation of microglia, as well as in the differentiation and development of neurons [16].

Recently, the role of TLRs in the AD pathological process has attracted the attention of investigators. Previous studies demonstrated that modulating TLRs results in changes in pathology and neurobehavioral functions in AD rodent models [17–20]. For example, a deficiency of TLR4 in AD models up-regulated cytokines and glial cell activation [21]. The activation of TLR4 by agonists improved cognitive impairments in rat models of AD [22]. TLR2, a member of the TLR family but different from TLR4 and other TLRs, is mediated through MyD88 alone. Recent studies demonstrated that activated bone-marrow-derived microglia (BMDM) could uptake Aβ and help to clear Aβ deposition [23], while TLR2 deficit BMDM could not perform its Aβ clearance function; moreover, TLR2 deficiency aggregated cognitive dysfunction in APP/PS1 transgenic mice [24, 25]. In contrast, other studies have reported that long-term administration of the TLR2 inhibitor in AD mice could reduce Aβ aggregation and glial activation [26], and that TLR2 gene knockout and the blocking of the interaction between TLR2 and MyD88 could attenuate the neurotoxicity and pathological changes of AD [27, 28]. While these contradictory phenomena could be interpreted as the consequence of different experimental conditions, such as differences in animal models, observed time points, and cell types, it is nonetheless apparent that TLR2 does play a role in the process of AD, although the exact effect remains to be elucidated.

To clarify the role of TLR2 in the pathological process of AD, in the present study, TLR2 knockout (KO) plus APPswe/PSEN1dE9 transgenic mice (AD-TLR2KO) were generated. Cognitive and emotional behavioral tests were conducted on the mice at the age of 12 months. Cortical thickness and white matter integrity were evaluated using brain magnetic resonance imaging (MRI). Neuron loss was evaluated using NeuN staining. Aβ, GFAP, proteins related to synaptic function, endogenous ligands for TLR2, and the activation of downstream signaling of TLR2 in brain tissue were detected by Western blots and immunohistochemistry (IHC) staining.

## RESULTS

### Genomic deletion of TLR2 induced cognitive impairment

**Figure 1:**
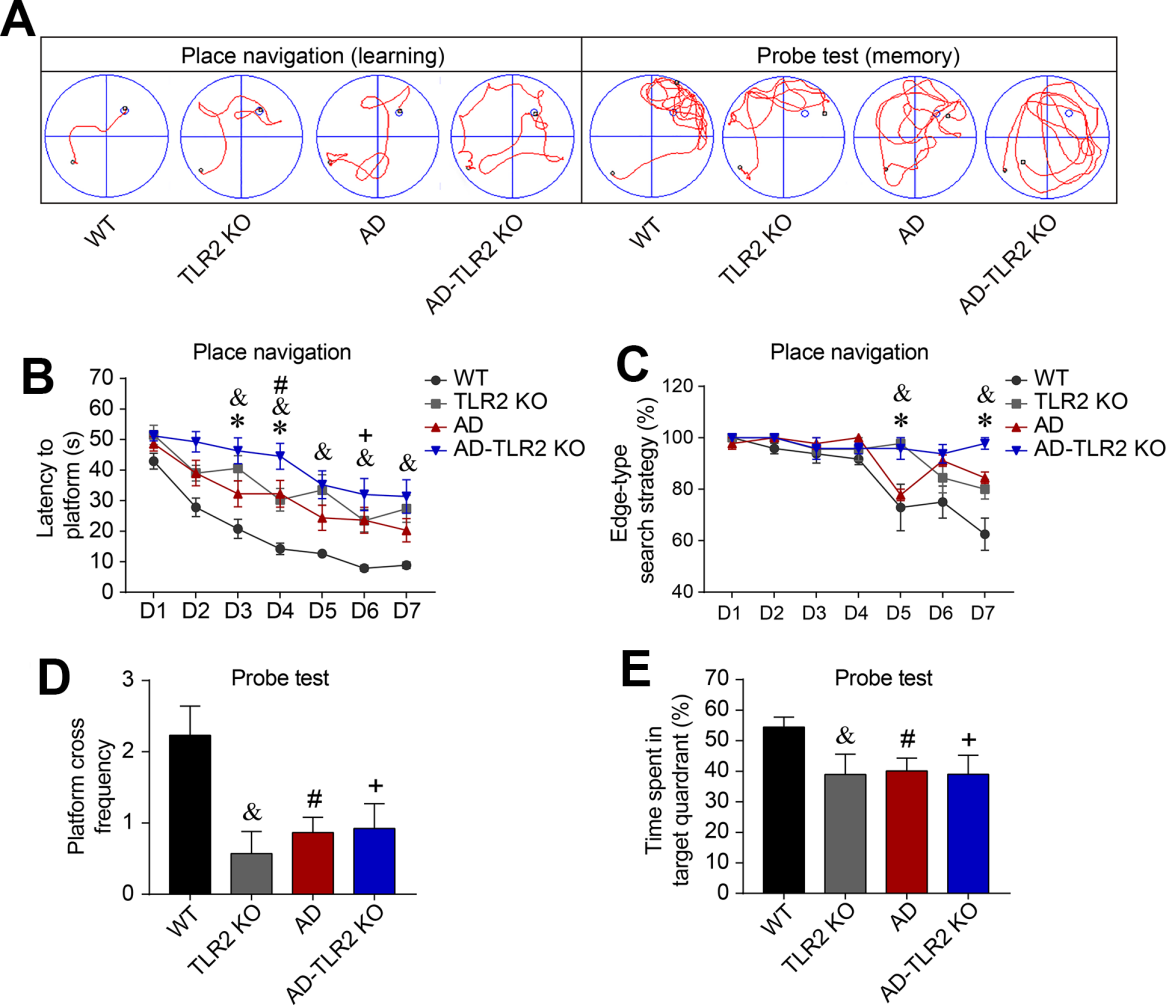
**Genomic deletion of TLR2 accelerated cognitive impairments in mouse models of AD.** Morris water maze tests (MWM) were performed on mice for seven consecutive days of place navigation processes, and a probe test was conducted on the 8^th^ day for mice aged 12 months. (**A**) Representative track plots of MWM for place navigation and probe tests. (**B**) The latency (time to find the hidden platform) increased in TLR2 knockout mice (&: TLR2KO vs. WT, p<0.05), AD mice (#: AD vs. WT, p<0.05), and AD-TLR2KO mice (+: AD-TLR2KO vs. WT, p<0.05). Moreover, the latency was prolonged in AD-TLR2KO mice compared with AD mice (*: AD-TLR2KO vs. AD, p<0.05). (**C**) Since mice have the habit of swimming along the pool, most of the mice searched the platform using an edge-type strategy on the first day of the test. As the tests progressed, WT mice converted to the tendency-type and straight-type searching strategies. However, compared with WT mice, AD mice exhibited a significantly higher ratio of the edge-type searching strategy (#: AD vs. WT, p<0.05). In addition, the ratio of the edge-type strategy in TLR2KO mice was significantly higher than that in WT mice (&: TLR2KO vs. WT, p<0.05). Importantly, the ratio of the edge-type strategy increased in AD-TLR2KO mice compared with AD mice (*: AD-TLR2KO vs. AD, p<0.05). (**D** and **E**) In the probe test, the frequencies of crossing the platform area in AD, AD-TLR2KO, and TLR2KO mice were significantly lower than those in WT mice (p<0.05, **D**). The time spent in the target quadrant among AD, AD-TLR2KO, and TLR2KO mice was significantly less than that among WT mice (p<0.05, **E**). However, there was no difference in the frequencies of crossing the platform area or in the time spent in the target quadrant between AD and AD-TLR2KO mice (Note: &: TLR2KO vs. WT, p<0.05; #: AD vs. WT, p<0.05; +: AD-TLR2KO vs. WT, p<0.05; *: AD-TLR2KO vs. AD, p<0.05. n=11~15/group).

Learning and memory function were detected at the age of 12 months. As shown in Figure 1A and 1B, the latencies (the time taken to find the hidden platform during the acquisition/learning phase) in AD mice were significantly longer than those in wild type (WT) control mice (p<0.05) from the 3^rd^ day to the 7^th^ day in the acquisition/learning phase. The latencies in TLR2KO mice were also significantly longer than those in WT control mice (p<0.05). Importantly, the latencies in AD-TLR2KO mice were significantly prolonged compared with those in AD mice (p<0.05) during the acquisition/ learning phase.

Since mice have the habit of swimming along the pool, most searched the platform using an edge-type strategy on the first day of testing. As the tests progressed, WT mice converted to the tendency-type and straight-type searching strategies. Compared with WT mice, AD mice demonstrated a significantly higher ratio of the edge-type searching strategy (p<0.05, Figure 1C). In addition, the ratio of the edge-type strategy in TLR2KO mice was significantly higher than that in WT mice (p<0.05, Figure 1C). Importantly, the ratio of the edge-type strategy increased in AD-TLR2KO mice compared with AD mice (p<0.05, Figure 1C).

In the probe test on the 8th day, the mice were placed into water from the 3rd quadrant, which was farthest from the platform (Figure 1A). The results showed that the frequencies of crossing the platform area in AD, AD-TLR2KO, and TLR2KO mice were significantly lower than those in WT mice (p<0.05, Figure 1D). In addition, the time spent in the target quadrant among AD, AD-TLR2KO, and TLR2KO mice was significantly less than that among WT mice (p<0.05, Figure 1E). There was no difference, however, in the frequencies of crossing the platform area or in the time spent in the target quadrant between AD and AD-TLR2KO mice.

The results indicated that TLR2 gene knockout significantly impaired learning ability and reference memory function. Moreover, TLR2 deficiency exacerbated learning impairment, but not reference memory function, in AD mice at the age of 12 months.

### Genomic deletion of TLR2 decreased spontaneous activity and increased anxiety and depression

**Figure 2:**
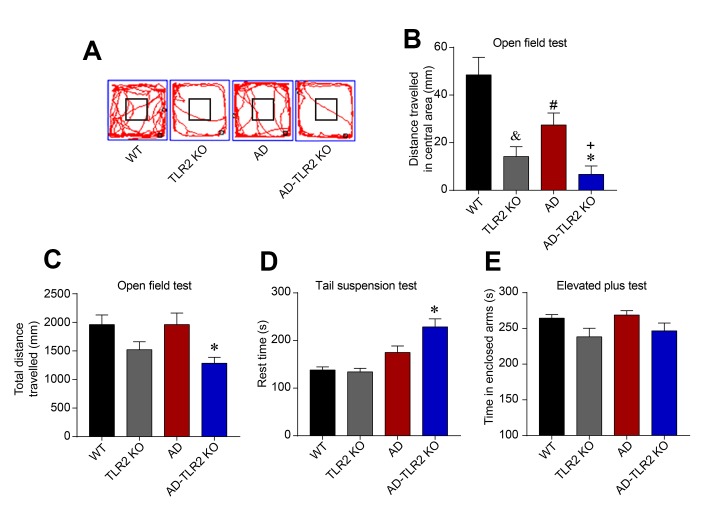
**TLR2 knockout increased the anxiety and depression states in mouse models of AD.** (**A**) Representative track plots from open field maze test. (**B**) The exploration times in the central area were significantly shorter in AD, TLR2, and AD-TLR2KO mice compared with WT mice (p<0.05). Moreover, the exploration time in the central area among AD-TLR2KO mice was shorter than that among AD mice (p<0.05). (**C**) The total traveled distance of AD-TLR2KO mice was shorter compared with WT mice (p<0.05). (**D**) In the tail suspension test, the rest time for AD-TLR2KO mice significantly increased compared with WT mice (p<0.05). (**E**) However, the time in enclosed arms did not show a significant difference among the groups in the elevated plus maze test (n=11~15/group).

In the open field test (Figure 2A), our data indicated that, compared with WT mice, the AD, TLR2, and AD-TLR2KO mice exhibited significantly shorter exploration times in the central area (p<0.05). The exploration time among AD-TLR2KO mice was shorter than that among AD mice (p<0.05, Figure 2B). In addition, the total traveled distance was shorter among AD-TLR2KO mice compared with WT mice (p<0.05, Figure 2C). Moreover, in the tail suspension test, the rest time among AD-TLR2KO mice significantly increased compared with that among WT mice (p<0.01, Figure 2D). The time in the enclosed arms did not show a significant difference among the groups in the elevated plus maze test (Figure 2E).

### Genomic deletion of TLR2 did not affect Aβ deposition in AD mice

**Figure 3:**
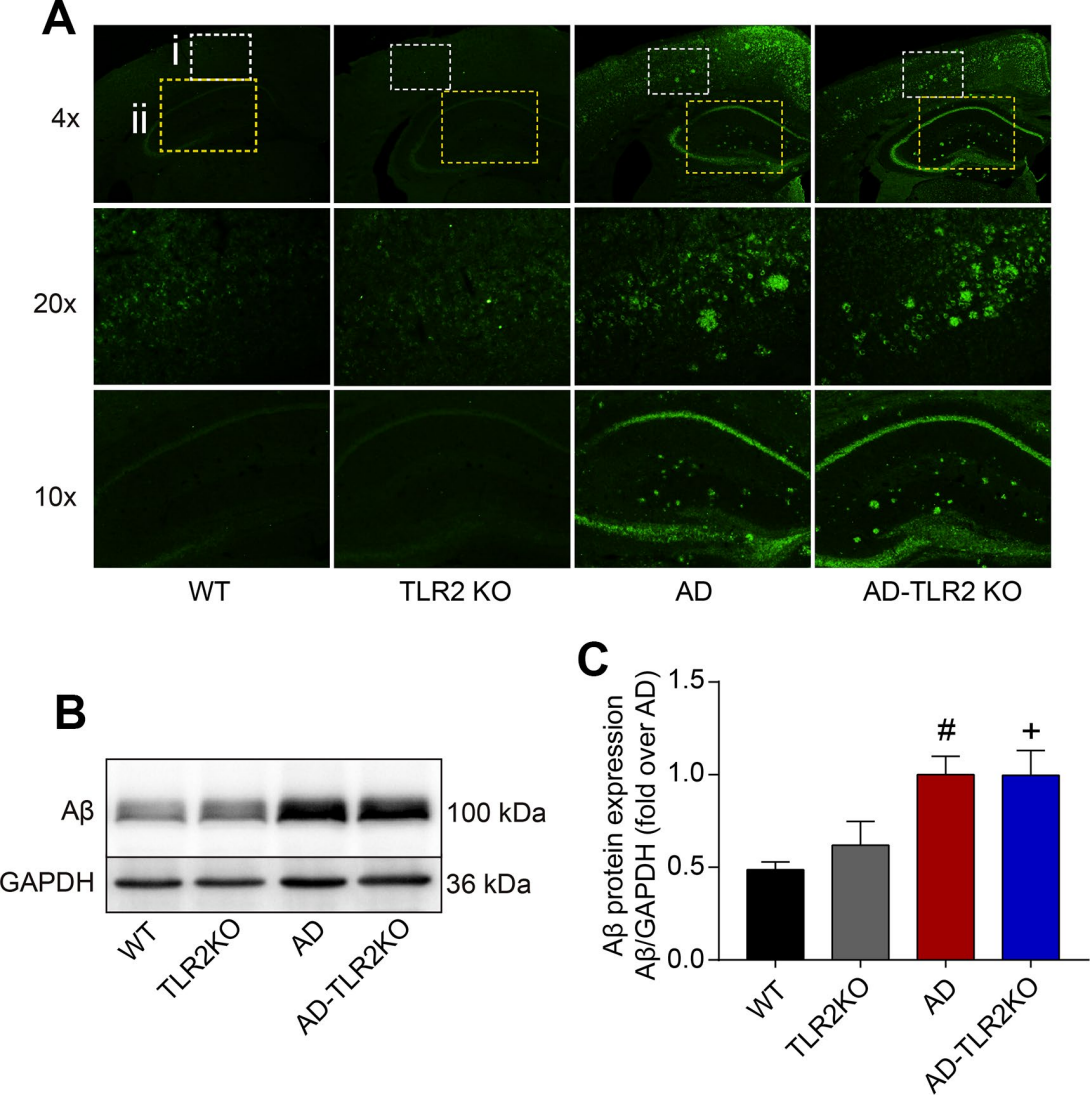
**Aβ deposition in mouse brains.** (**A**) Representative immunofluorescence images of Aβ deposition in mouse brains (4x, 20x: cortex; 10x: hippocampus). (**B**) Representative Aβ levels in brain tissues detected by Western blots. (**C**) Results from quantitative analyses of Western blots showed that Aβ levels were significantly higher in AD and AD-TLR2KO mice compared with WT and TLR2KO mice, respectively (#: AD vs. WT, p<0.05; +: AD-TLR2KO vs. WT, p<0.05). However, there was no significant difference in Aβ levels between AD and AD-TLR2KO mice (n=6 / group).

The results from Western blots and immunofluorescence staining, as shown in Figure 3, demonstrated abundant Aβ deposition in the cortex and hippocampus in AD and AD-TLR2KO mice brains. The levels of Aβ were significantly higher in AD and AD-TLR2KO mice compared with those in WT and TLR2KO mice, respectively (p<0.05). However, there was no difference in Aβ levels between AD and AD-TLR2KO mice.

### Genomic deletion of TLR2 accelerated white matter damage in AD mice

**Figure 4:**
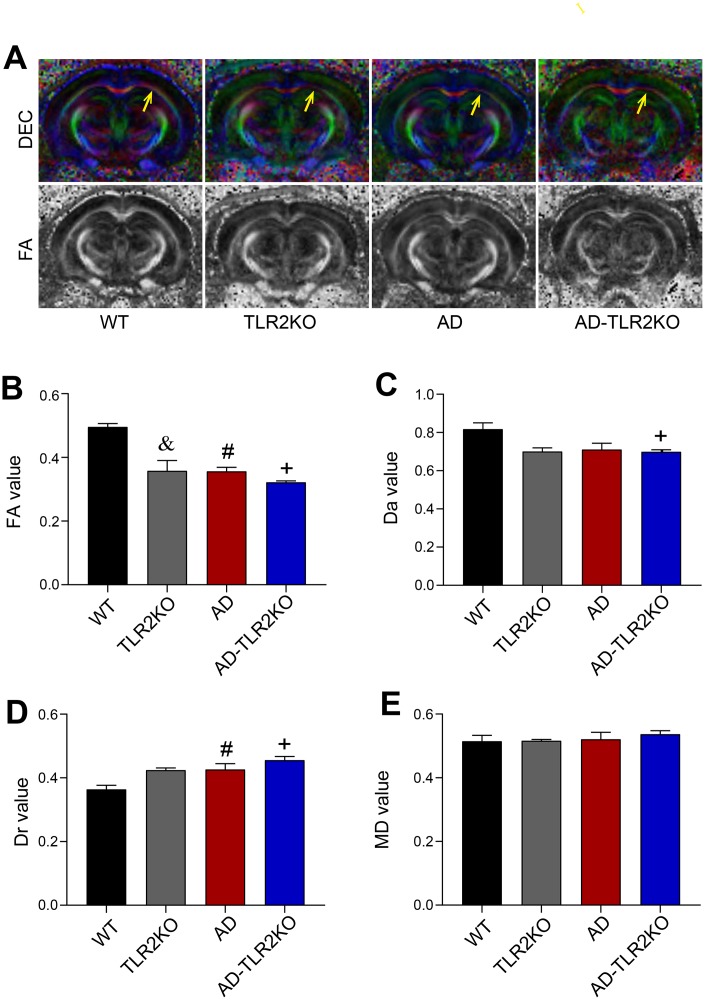
**White matter integrity evaluated by DTI images detected by 7 Tesla MRI system.** (**A**) Representative diffusion-encoded-color (DEC) map and fractional anisotropy (FA) map showing white matter injury (yellow arrow). (**B**) Quantitative analysis showed that the FA value significantly decreased in TLR2KO mice (&), AD mice (#), and AD-TLR2KO mice (+) compared with WT mice (p<0.05, **B**). (**C**) Axial diffusivity (Da) value decreased in AD-TLR2KO mice compared with WT mice (+: p<0.05). (**D**) Radial diffusivity (Dr) value increased in AD (#) and AD-TLR2KO (+) mice compared with WT mice (p<0.05). (**E**) There was no significant deference in the mean diffusivity (MD) value among the groups. (n= 4~6 / group).

**Figure 5:**
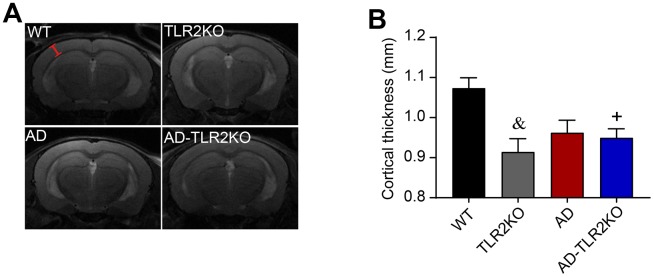
**Thickness of cortex evaluated by T2-weighted images detected by 7 Tesla MRI system.** The cortical thickness was measured by Image J software on T2-weighted images obtained from Paravision software. (**A**) Representative T2-weighted images used for measurement of cortical thickness (red line segment indicates the measured regions). (**B**) Results showed that cortical thickness was reduced in TLR2KO mice and AD-TLR2KO mice compared with WT mice (&: TLR2KO vs. WT, p<0.05; +: AD-TLR2KO vs. WT, p<0.05). (n= 4~6 / group).

By using a 7 Tesla MRI system, the WMD in mice brains was evaluated at the age of 12 months. After MRI scanning, diffusion tensor imaging (DTI) maps were obtained (Figure 4A). Fractional anisotropy (FA), axial diffusivity (Da), mean diffusivity (MD), and radial diffusivity (Dr) values were analyzed, and quantitative analysis of the same region of interest (ROI) for each mouse was performed using DSI studio software. As shown in Figure 4A, the FA value in the external capsule (EC) area, indicating the integrity of white matter, declined in AD and AD-TLR2KO mice compared with WT and TLR2KO mice (p<0.05, Figure 4B). The values of Da and Dr were also associated with myelin and axonal damage. The data showed that the Da value decreased and the Dr value increased in AD-TLR2KO mice compared with WT mice (p<0.05, Figure 4C, 4D). In addition, cortical thickness was measured by Image J software on T2-weighted images obtained from Paravision software (Figure 5A). The results demonstrated that the thickness of the cortex was significantly reduced in TLR2KO mice (p<0.05, Figure 5B) compared with WT mice.

### Genomic deletion of TLR2 increased glial activation in mouse models of AD

**Figure 6:**
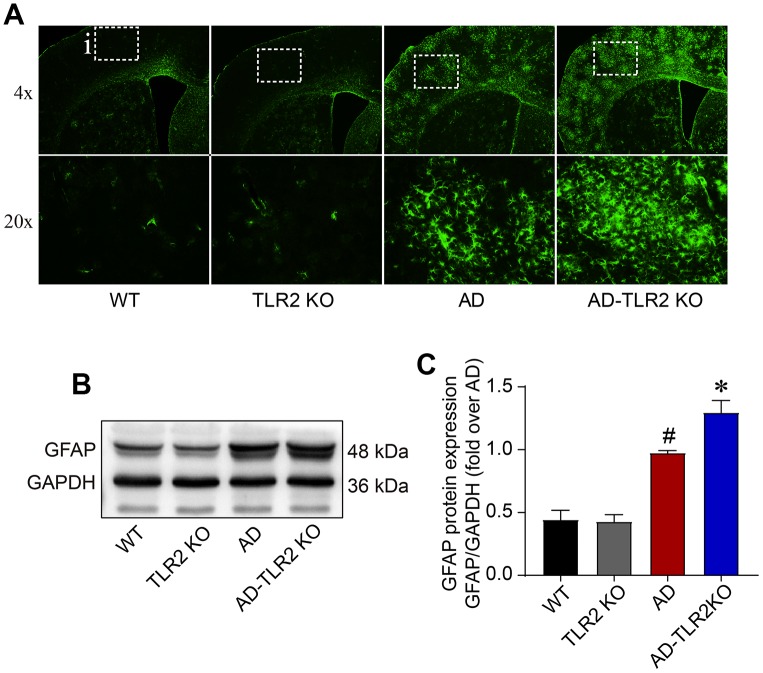
**Levels of GFAP in mouse brains.** (**A**) Representative immunofluorescence staining of GFAP, a marker of astrocytes. (**B**) Representative bands of GFAP in brain tissues detected by Western blots. (**C**) GFAP significantly increased in AD mice and AD-TLR2KO mice compared with WT and TLR2 mice, respectively (#: AD vs. WT, p<0.05; +: AD-TLR2KO vs. WT, p<0.05). Moreover, the level of GFAP in AD-TLR2KO mice was significantly higher than that in AD mice (*: AD-TLR2KO vs. AD, p<0.05). (n= 6 / group).

Glial fibrillary acidic protein (GFAP), a specific marker for astrocytes, was detected using Western blots and immunofluorescence staining. As shown in Figure 6, the level of GFAP significantly increased in AD mice and AD-TLR2KO mice compared with WT mice (p<0.05). Moreover, the GFAP level was higher in AD-TLR2KO mice than in AD mice (p<0.05).

### Genomic deletion of TLR2 did not significantly affect the proteins associated with synaptic plasticity

**Figure 7:**
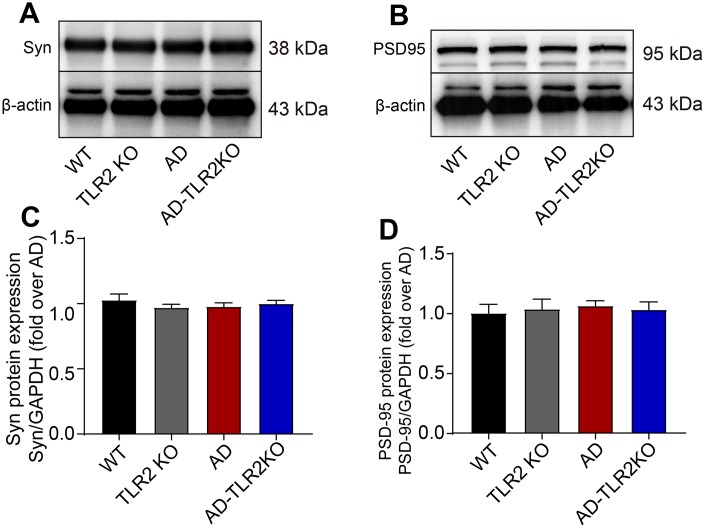
**Levels of synaptophysin (Syn) and PSD95 in mouse brains.** (**A**) Representative image of Western blots for Syn. (**B**) Representative image of Western blots for PSD95. (**C**, **D**) There was no significant difference in the levels of Syn (**C**) and PSD95 (**D**) between AD and AD-TLR2KO mice. (n= 6 / group).

Previous research has reported that the synaptic plasticity in AD model was damaged, which might be one explanation for the acceleration of memory deficits and disease processes. In the present study, the protein levels of synaptophysin (Syn) and postsynaptic density protein 95 (PSD95) were detected using Western blot (Figure 7). The results showed no significant differences in Syn and PSD95 protein levels among each group of 12-month-old mice.

### Genomic deletion of TLR2 induced neuron loss in mouse brains

**Figure 8:**
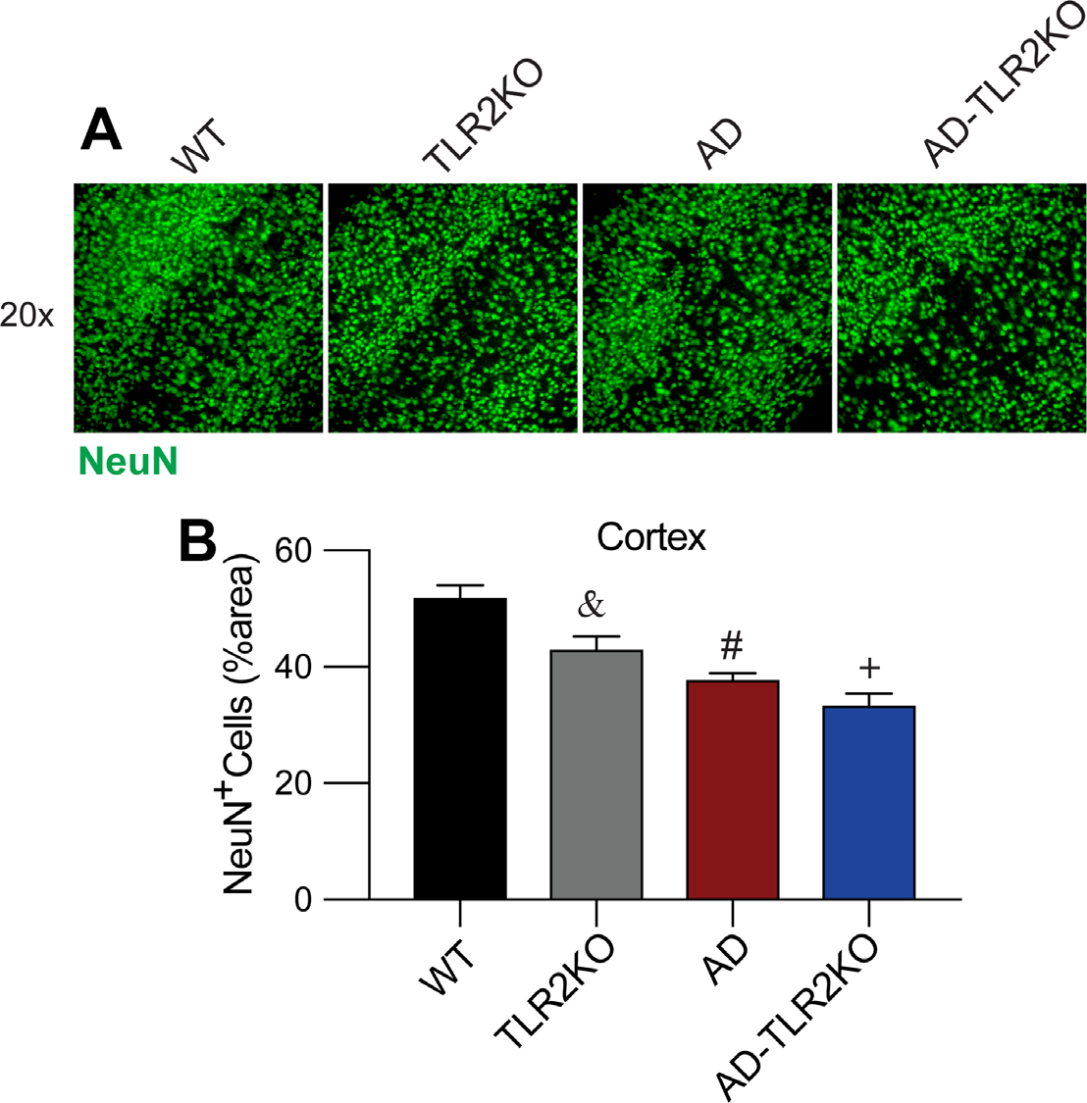
**TLR2 deficiency resulted in neuronal loss in mouse brains.** (**A**, **B**) Neuronal density detection and quantification analysis indicated a lower capacity of NeuN^+^ cells in AD, TLR2KO, and AD-TLR2KO mice when compared with WT mice (n=6 for each group, p<0.05).

The neuronal number was evaluated at the age of 12 months by IHC staining for NeuN, a marker for neurons. As shown in Figure 8, neuronal numbers in TLR2KO mice significantly decreased compared with those in WT mice (p<0.05). In addition, neuronal numbers were also lower in AD mice and AD-TLR2KO mice compared with those in WT mice (p<0.05). However, no significant difference in neuronal number was observed among AD, TLR2KO, and AD-TLR2KO mice (p>0.05).

### Genomic deletion of TLR2 increased the protein level of biglycan, but not HMGB1, in AD mice

**Figure 9:**
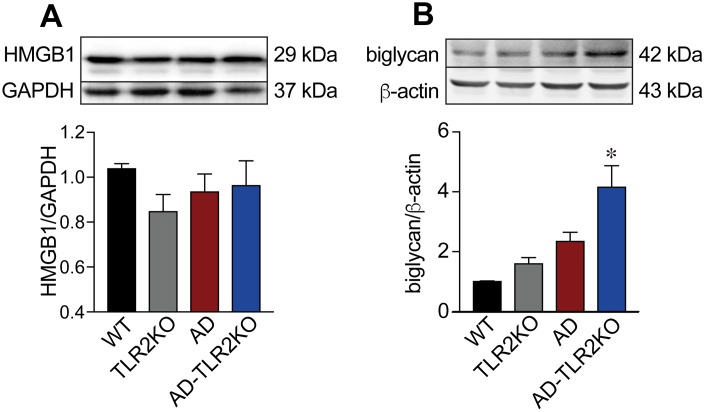
**Expression of endogenous ligands for TLR2.** (**A**) Expression of biglycan in AD-TLR2KO mice increased significantly compared with that in WT, AD, and TLR2KO mice (p<0.05). (**B**) HMGB1 in the four groups did not show a significant difference (p>0.05).

Biglycan and high mobility group box 1 (HMGB1), endogenous ligands for TLR2, were detected using Western blot. As shown in Figure 9, the protein level of biglycan in AD-TLR2KO mice was significant higher that that in WT, TLRKO, and AD mice (p<0.05). However, there was no significant difference in HMGB1 among the four groups at the age of 12 months (p>0.05).

### Genomic deletion of TLR2 decreased the activation of TLR-MyD88-NFκB signaling in AD mose brains

**Figure 10:**
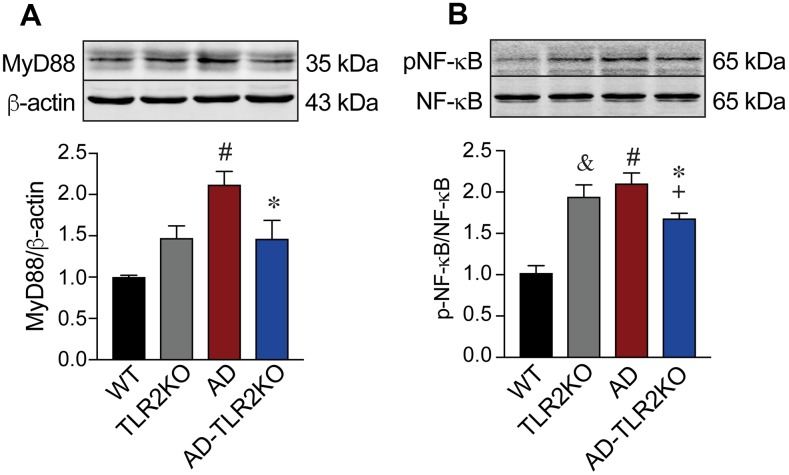
**Activation of MyD88-NFκB signaling.** Expression of MyD88 significantly increased in AD mice compared with WT mice (p<0.05). The increased MyD88 was inhibited in AD-TLR2KO mice compared with AD mice (p<0.05). Expression of pNF-kB significantly increased in AD mice compared with WT mice (p<0.05). The phosphorylation of NF-kB was inhibited in AD-TLR2KO mice compared with AD mice (p<0.05).

MyD88 is a key adaptor protein in TLR-mediated signaling pathways. Activation of TLR2 mediates MyD88 alone, leading to the phosphorylation of NFκB and the regulation of inflammatory responses. As shown in Figure 10, MyD88 and p-NFκB significantly increased in the brains of AD mice (p<0.05), but were inhibited in AD-TLR2KO mice (p<0.05).

## DISCUSSION

Prior research has shown that AD is a heterogeneous disease involving multiple pathogenic factors, including β-amyloid deposition, tau-protein hyperphosphorylation, and inflammatory reaction, among other mechanisms [29], of which immune inflammatory response is the core pathological mechanism. The imbalance of immune inflammatory response and its regulatory mechanism promotes the occurrence and development of AD. Many immune inflammatory factors are involved in AD [30], including microglia, peripheral immune cells, the interleukin family, the tumor necrosis factor family, and TLR-mediated transduction pathways [7, 8, 13, 31, 32].

TLRs are a type I transmembrane receptor superfamily that plays an important role in the induction and regulation of immune inflammatory responses. At present, at least 13 TLRs have been found in mammals, including 10 TLRs (TLR1 to TLR10) in humans and 12 in mice (TLR1 to TLR9, TLR11 to TLR13) [33]. Activated by their ligands, TLRs activate downstream protein kinases through regulatory proteins, leading to the activation of nuclear transcription factors (NFκB) and/or the interferon regulatory factor (IRF), as well as to the release of immune inflammatory factors [33, 34]. TLR signaling is mainly mediated by two regulatory proteins, MyD88 (myeloid differentiation factor 88) and TRIF (TIR-domain-containing adapter-inducing interferon-β), which are, respectively, mediators of the MyD88-dependent signaling pathway and the TRIF-dependent (MyD88-independent) signaling pathway.

Recently, the role and mechanism of TLRs in the development of AD, and the role of specific drugs for TLRs in AD, have been reported [35]. TLR2, TLR4, TLR5, TLR7, and TLR9 mRNAs were found to be significantly up-regulated in the plaque-associated brain tissue of aged APP23 transgenic mice compared to the plaque-free tissue [36]. Expressions of mRNAs and proteins of TLR2 and TLR4 in peripheral blood mononuclear cells (PBMCs) were markedly elevated in late-onset AD (LOAD) patients [37]. Levels of TLR4-dependent cytokines, such as tumor necrosis factor (TNF)-alpha, interleukin (IL)-1beta, IL-10, and IL-17, in the brains of transgenic AD mice were significantly higher than those in non-transgenic WT mice [38]. Behavioral, molecular, and electrophysiological evidence indicates that early minor stimulation of microglial TLR2 and TLR4 receptors attenuates AD-related cognitive deficits in rats [39]. These data strongly suggest that TLR signaling is involved in AD progression and can be a new therapeutic target for AD.

TLR2, different from other TLRs, is only propagated through the MyD88-dependent pathway. Previous studies have indicated that mice lacking TLR2 receptors are largely protected against ethanol-induced cytokine and chemokine release, as well as against behavioral-associated effects during alcohol abstinence [40]. Activation of TLR2 by intracerebroventricular injection of its ligand, Pam3CSK4, induced sickness behavior symptoms, including anorexia, hypoactivity, and hyperthermia [41]. Recent studies on the role of TLR2 in AD have yielded conflicting results. For example, one study revealed that inhibiting TLR2 by the anti-TLR2 antibody improved performance in spatial learning by decreasing microglial and astroglial activation, and by reducing Aβ plaque burden [26]. However, another study reported that the intracerebroventricular administration of low-dose TLR2 ligands reduced disturbances in spatial and working memory and reversed the impaired long-term potentiation induced by Aβ [39].

To further clarify the effect of TLR2 on neurobehavioral and pathological changes in AD, in the present study, we used APPswe/PSEN1dE9 transgenic (AD, App^+^Psen1^+^) mice and TLR2 gene knockout (TLR2KO, Tlr2^-/-^) mice to generate Tlr2^-/-^App^+^Psen1^+^ (AD-TLR2KO) mice. Neurobehavior functions in the mice were evaluated with different gene types at the age of 12 months. The data from the Morris water maze (MWM) demonstrated that the genomic deletion of TLR2 alone prolonged the latencies in finding the hidden platform during the acquisition/learning phase, decreased the frequencies of crossing the platform area, reduced the time spent in the target quadrant, and increased the ratio of the edge-type strategy. Moreover, the genomic deletion of TLR2 prolonged the latencies in finding the hidden platform during the acquisition/learning phase and increased the ratio of the edge-type strategy. The results indicated that the TLR2 deficit alone impaired learning ability and reference memory function, and that this deficit also exacerbated learning impairment, but not reference memory function, in AD mice at the age of 12 months. Open field tests demonstrated that TLR2KO decreased exploration times in the central area among AD mice as well as the total traveled distance. In the tail suspension test, the rest time significantly increased in AD-TLR2KO mice when compared with WT mice. These data indicate that the genomic deletion of TLR2 significantly aggravated the increased anxiety and depression in AD mice at the age of 12 months.

Diffusion tensor imaging (DTI) is a vital, non-invasive method for characterizing microstructural changes or differences with neuropathology and treatment [42, 43]. To evaluate brain damage, in the present study, diffusion tensor images were obtained and analysis of selected ROIs from ECs was performed by using a 7.0-T BioSpec 70/20 MRI scanner and diffusion spectrum magnetic resonance imaging (DSI) studio post-analysis software. The FA value represents brain white matter integrity, which is highly sensitive to microstructural changes. Da and Dr are indicators of myelination and axonal damage. The mean diffusivity (MD) value may serve as a marker of tissue water in edema and proliferation in neoplasia [42, 44, 45]. Our results demonstrated that the FA value significantly decreased in TLR2KO, AD, and AD-TLR2KO mice. In addition, the values of Da decreased, while those of Dr increased, in AD-TLR2KO mice compared with WT mice. The cortical thickness measured from T2-weighted images decreased in TLR2KO and AD-TLR2KO mice compared with WT mice. These data indicate that the TLR2 deficit was involved in WMD, myelination and axonal impairment, and brain atrophy, and further aggravated WMD in AD mice. Impaired WMD might be a mechanism by which the TLR2 deficit induces neurobehavioral dysfunction.

Neuropathology in AD is characterized by the deposition of Aβ, senile plaques, and neurofibrillary tangles (NFTs). Gliosis and increased inflammation play important roles in the disease’s pathogenesis. A previous study demonstrated that low-dose TLR2 ligands decreased Aβ deposits [39]. Conversely, other studies reported that blocking TLR2 with the anti-TLR2 antibody reduced Aβ plaque burden in APP/PS1 mice [26], and that the disruption of TLR2–MyD88 interaction inhibited hippocampal glial activation and reduced Aβ burden in 5XFAD mice [28]. To understand the mechanisms underlying the effect of the TLR2 deficit on impaired neurobehavioral functions, we detected Aβ deposition in mouse brains. Our data showed that Aβ deposition significantly increased in AD, but not in TLR2KO, mice. The increased Aβ deposition in AD mice was not inhibited in AD-TLR2KO mice. The data indicated that the TLR2 deficit resulted in the impairment of neurobehavioral functions, possibly via a non-Aβ mechanism.

Glial activation and inflammatory reaction are important self-protection mechanisms [8]. However, excessive inflammatory response is detrimental and accelerates disease process [46]. Many studies have shown excessive astrocyte gliosis and inflammation in patients with AD and in animal models of AD [31, 47]. Our data demonstrated that GFAP, a marker for astrocytes, significantly increased in AD mice compared with WT mice. Moreover, the increased GFAP was enhanced in AD-TLR2KO mice. The data indicated that the TLR2 deficit increased GFAP activation in AD mice, which might be a mechanism by which the TLR2 deficit induces the impairment of neurobehavioral functions.

Previous studies have reported that synaptic function is impaired in AD [48–50]. To investigate the effect of TLR2 on synaptic plasticity, we detected the protein levels of postsynaptic density 95 (PSD-95) and synaptophysin (Syn) in brain tissue. The results demonstrated no difference in the levels of PSD-95 and Syn among the groups, which in turn indicates that TLR2 deficiency does not lead to the changes in proteins associated with synaptic plasticity in mice at the age of 12 months.

One of the most important pathological characteristics in AD brains is the loss of neurons. In the present study, neuronal numbers were evaluated in the brains of mice at the age of 12 months by IHC staining for a neuronal marker, NeuN. The results showed (Figure 8) that neuronal numbers significantly decreased in TLR2KO, AD, and AD-TLR2KO mice compared with WT mice (p<0.05). However, no further decreased neuronal numbers were observed in AD-TLR2KO mice compared with AD mice (p>0.05). These data indicate that TLR2 deficiency was not able to induce the loss of neurons in AD brains. In contrast, TLR2 deficiency alone induced the loss of neurons in brains without AD.

The classic hallmarks of AD include the formation of Aβ plaque deposits and NFTs. However, the mechanisms underlying the initiation and progression of AD remain unclear. Recent studies have demonstrated that endogenous danger signals stimulate innate immune cells via TLR2 and TLR4 and accelerate inflammatory responses [51]. Biglycan, one prototype extracellular matrix-derived damage-associated molecular pattern, functions as an endogenous agonist of TLR2/4 and mediates sterile inflammation through TLR2- and/or TLR4-dependent signaling pathways [52]. Another central endogenous inflammatory mediator, HMGB1, when interacting with TLR signaling pathways, contributes to the pathogenesis of several inflammatory disorders [53]. However, our data failed to show increased protein levels of biglycan and HMGB1 in AD mice compared with WT mice. This phenomenon could be interpreted as meaning that since the mice used in the present study were 12 months old, some biochemical reactions occurring in the early stage of the disease are not observable in the late stage. Interestingly, TLR2 deficiency significantly increased the protein level of biglycan, but not HMGB1, indicating that TLR2 might play an important role in the clearance of biglycan.

TLR2 signaling is mediated via the MyD88-dependent pathway, leading to the phosphorylation of NFκB and to inflammatory responses. Previous studies reported that the expression of TLRs was up-regulated and TLR-mediated inflammation was activated in AD brains [36–38]. Our data, showing increased protein levels of MyD88 and p-NFκB in AD mice at the age of 12 months, indicate that the TLR downstream MyD88-dependent pathway was activated in AD brains. In addition, increased MyD88 and p-NFκB were inhibited in AD-TLR2KO mice, indicating that, in the late stage of AD, the activation of MyD88-dependent signaling is mediated through TLR2.

In summary, the present study demonstrated that the genomic deletion of TLR2 induced impaired neurobehavioral function, WMD and loss of neurons, brain atrophy, and the activation of astrocytes. TLR2 deficiency exacerbated impaired neurobehavioral function and enhanced the activation of astrocytes in AD brains without affecting Aβ deposition. Further studies on molecular mechanisms, especially in the early stage of AD, must be conducted. In addition, since TLR signaling is involved in the pathology of AD, it has been considered as a therapeutic target for AD. Several studies have demonstrated that the activation or inhibition of TLR2 by an exogenous reagent could attenuate impaired neuronal function and pathological changes in AD models. However, the reported results have been controversial. Therefore, the exact effect of TLR2 agonists/antagonists on neurobehavioral functions and pathological changes in AD, their dose–effect relationship, and the time window for treatments require further investigation.

## MATERIALS AND METHODS

### Animal generation and experimental design

APPswe/PSEN1dE9 transgenic (AD, App^+^Psen1^+^) mice and TLR2 knockout (TLR2KO, Tlr2^-/-^) mice were purchased from the Animal Model Research Center of Nanjing University, China. Step 1 involved breeding TLR2KO and AD mice together to generate Tlr2^+/-^App^+^Psen1^+^ mice and Tlr2^+/-^ mice. Step 2 combined Tlr2^+/-^App^+^Psen1^+^ to generate Tlr2^-/-^App^+^Psen1^+^ (AD-TLR2KO) mice. Meanwhile, Tlr2^+/-^ mice were mated with each other to generate Tlr2^-/-^ (TLR2KO) mice and Tlr2^+/+^ (WT) mice. Step 3 involved breeding App^+^Psen1^+^ mice with each other to generate App^+^Psen1^+^ (AD) mice. The 3^rd^ generation of the mice was used in our experiment. All the mice were bred at the Experimental Animal Center of Xuzhou Medical University and were kept in the same housing conditions (temperature, 22–23°C; 12h light-dark cycle; food and water were available ad libitum). All aspects of animal care and experimental protocols were approved by the Xuzhou Medical University Committee on Animal Care.

### Morris water maze (MWM)

The MWM system (Zhenghua Biological Instruments, China) was used to evaluate spatial learning and reference memory according to the processes described previously in our laboratory [33]. Briefly, the inner diameter of the circular pool was set at 122cm, and the depth was set at 40cm. All sides of the pool were painted white to create a strong contrast with the mice. A circular platform with a rough white surface (diameter, 6cm) was placed in the middle of the 1^st^ quadrant (target quadrant) and immersed 1cm below the water surface. Four different black signs were settled in the middle side of each quadrant as spatial reference cues. On the 1^st^ to 7^th^ days (place navigation), each mouse was given four trials per day (60 seconds per trial) with a 10-minute rest time between trials. During the acquisition phase, the platform was fixed in the same location. Mice were released into the water from four directions in a random order each day. When a mouse succeeded in arriving on the platform within 60 seconds, it was permitted to remain on the platform for 10 seconds. If a mouse failed to reach the platform in 60 seconds, it would be guided onto the platform and allowed to remain there for 10 seconds. Latency to arrive onto the platform (latency to platform) and search strategy were recorded. On the 8^th^ day, a probe trial was given at 24 hours after the last acquisition day. To perform the probe trial, the platform was removed from the water and the mice were allowed to swim for 60 seconds to search for the platform. The time spent crossing the platform site and the time spent in the target quadrant were recorded by MWM system software.

### Open field maze (OFM)

Open field mazes are commonly used to evaluate spontaneous locomotor activity and exploratory behavior. In the present study, an open field test was performed as previously described [54]. Briefly, the open field system (Zhenghua Biological Instruments, China) includes four white Plexiglas (50×50×50 cm) open-field arenas, video acquisition, and an analysis system. During the test, mice were placed at one side of the arena and recorded for five minutes. Total traveled distance (mm), total time spent in the central zone, and resting time in the opening field were recorded.

### Elevated plus maze (EPM)

The elevated plus maze (EPM) was performed to assess anxiety and activity among the mice according to a previously published method [55]. Briefly, the EPM system consists of two open arms and two enclosed arms with an open roof. The maze was elevated 40cm from the floor. During the experiment, mice were placed at extremity facing the walls of the enclosed arm and were allowed to explore the maze freely for five minutes. Total traveled distance, central entry frequency, and rest time in the maze were recorded using a video analysis system (Zhenghua Biological Instruments, China).

### Tail suspension test (TST)

A tail suspension test (TST) was evaluated for depression behavior as previously described [56]. Briefly, mice were suspended in an inescapable but moderately stressful situation. They were suspended 40cm above the floor using a nylon string taped to the tip of their tails. By using a video analysis system (Zhenghua Biological Instruments, China), the rest time during a six-minute period was recorded. A lack of escape-related behavior was considered as immobility and depression.

### MRI

An MRI was performed according to a previously reported process [57, 58]. Briefly, 12-month-old mice received MRI scans on a 7.0-T BioSpec 70/20 MRI scanner (Bruker BioSpin, Germany) in a Shanghai cancer institute (Shanghai, China). For MRI handling procedures, all mice were anesthetized with 1.5% isoflurane (R510-22, RWD Life Science Co., China) with a mixture of 70% nitrogen and 30% oxygen. Respiration and body temperature were monitored during MRI scanning. T2-weighted images (T2WI): Three orthogonal multi-slice turbo rapid-acquisition with relaxation enhancement (RARE) were acquired to render slice-positioning uniform (slice thickness: 0.7mm; repetition time: 2500ms; echo time: 35ms; imaging frequency: 300.3206893ms; imaged nucleus: 1H; matrix: 256x256). All T2WI images were reconstructed by Paravision 6.0.1 and analyzed in Image J software to measure the thickness of the cortex. Diffusion images (DTI) were acquired using a “Bruker: DtiEpi SpinEcho” sequence (TE=0.68267ms, and TR=2800ms). The diffusion time was 12ms. The diffusion encoding duration was 5ms. A DTI diffusion scheme was used, and a total of 30 diffusion sampling directions were acquired. The b-value was 2040.17s/mm^2^. The in-plane resolution was 0.140625mm. The slice thickness was 0.8mm. The diffusion tensor was calculated. By using DSI studio software, we measured the ROIs in the corpus callosum (CC) and external capsule (EC) regions to obtain fractional anisotropy (FA), axial diffusivity (Da), mean diffusivity (MD), and radial diffusivity (Dr) values.

### Immunohistochemistry (IHC) staining

IHC staining was performed according to a previously reported process in our laboratory [15]. Briefly, mice were transcardially perfused with phosphate buffer saline (PBS) followed by 4% paraformaldehyde (PFA). The brains were post-fixed in 4% PFA for 24 hours and dehydrated in 30% sucrose for 72 hours. Then, the brains were cut into coronal sections (25μm thickness) in a cryostat and stored in Cryoprotectant. The brain sections were washed with PBS and 0.3% PBST (0.3% Triton X-100 in PBS) and then blocked with 5% goat serum in 0.3% PBST for 2 hours at room temperature. Brain sections were then incubated with primary antibodies for Aβ (anti-beta amyloid, 60342-1-Ig, Proteintech), GFAP (anti-GFAP, ab7260, Abcam), and NeuN (anti-NeuN, ab104224, Abcam) overnight at 4°C. After washing with 0.3% PBST, the sections were incubated with Alexa Flour® 488 secondary antibody for two hours at room temperature.

### Western blot

Western blot was performed according to a previously reported process [34]. Briefly, the brain samples from four groups (n=6/per group) were collected, and then the proteins were extracted. A sodium dodecyl sulfate polyacrylamide gel (SDS-PAGE) electrophoresis system was used to separate the proteins, which were transferred onto 0.45-μm PVDF membranes (Millipore IPVH00010, MA, USA). The PVDF membranes were incubated with primary antibodies at 4°C overnight. After washing with washing buffer, the PVDF membranes were incubated with peroxidase-conjugated secondary antibodies. Signals were detected with a High-sig ECL Western Blotting Substrate (Tanon, Shanghai, China) and scanned with Bio-Rad ChemiDoc™ imaging systems. The primary antibodies used in this study were anti-Aβ (60342-1-Ig, Proteintech), anti-GFAP (ab7260, Abcam), anti-PSD95 (20665-1-AP, Proteintech), anti-Syn (17785-1-AP, Proteintech), anti-p-NFκB (#3033, Cell Signaling Technology), NFκB (#8242, Cell Signaling Technology), MyD88 (SAB3500472, Sigma-Aldrich), biglycan (ab58562, Abcam), HMGB1 (NB100-2322, Novus Biologicals), and anti-actin (ab822387, Abcam).

### Statistical analysis

Data are presented as mean ± SEM. Statistical analyses were performed using GraphPad Prism 7.0. Comparison between two experimental groups was analyzed by a two-tailed t-test. Differences among multiple time spots were based on one or two-way ANOVA followed by the Sidak post hoc correction. The significance level was set to 0.05.
